# A Geometric Morphometric Approach to the Analysis of Lip Shape during Speech: Development of a Clinical Outcome Measure

**DOI:** 10.1371/journal.pone.0057368

**Published:** 2013-02-25

**Authors:** Hashmat Popat, Stephen Richmond, Alexei I. Zhurov, Paul L. Rosin, David Marshall

**Affiliations:** 1 Applied Clinical Research and Public Health, School of Dentistry, Cardiff University, Cardiff, United Kingdom; 2 Cardiff School of Computer Science and Informatics, Cardiff University, Cardiff, United Kingdom; Boston Children's Hospital, United States of America

## Abstract

Objective assessments of lip movement can be beneficial in many disciplines including visual speech recognition, for surgical outcome assessment in patients with cleft lip and for the rehabilitation of patients with facial nerve impairments. The aim of this study was to develop an outcome measure for lip shape during speech using statistical shape analysis techniques. Lip movements during speech were captured from a sample of adult subjects considered as average using a three-dimensional motion capture system. Geometric Morphometrics was employed to extract three-dimensional coordinate data for lip shape during four spoken words decomposed into seven visemes (which included the resting lip shape). Canonical variate analysis was carried out in an attempt to statistically discriminate the seven visemes. The results showed that the second canonical variate discriminated the resting lip shape from articulation of the utterances and accounted for 17.2% of the total variance of the model. The first canonical variate was significant in discriminating between the utterances and accounted for 72.8% of the total variance of the model. The outcome measure was created using the 95% confidence intervals of the canonical variate scores for each subject plotted as ellipses for each viseme. The method and outcome model is proposed as reference to compare lip movement during speech in similar population groups.

## Introduction

The study of lip shape during speech has an important role in visual speech recognition among other related disciplines. The shape of the lips during speech has three important functions [1]. Firstly they are a place of closure for a number of phonemes such as /p/ and /b/. Secondly they can alter the size and shape of the oral cavity to differentiate /u/ from /i/ by lip protrusion. Finally they can act as a sound source where air passes through the space between the upper incisors and the lower lip under pressure causing friction during /f/. Speech readers demonstrate that information conveyed visually during the process of speech allows recognition of what is being said. Indeed, lip movement is known to play an important role in both sign language and communication between the deaf [2]. Adequate visibility of the face and distinct lip shapes aid speech perception and can help disambiguate speech sounds that can be confusable from acoustics alone, e.g., the unvoiced consonants /p/ (a bilabial) and /k/ (a velar) [3]. It is therefore clear that lip shape plays a significant role in verbal communication.

Disorders of speech such as dysarthrias can result from a physical or neurological deficit of the motor-speech system, of which the lips can be affected. Although the treatment for these conditions will depend on the effect the dysarthria has on control of the articulators, aims of rehabilitation will involve strengthening and increasing control over the articulator muscles and learning the correct mouth movements for phonemes. Objective assessments can be beneficial in these situations to allow the clinician to diagnose, treatment plan and quantitatively monitor change/outcome over time. In allied clinical specialities such as orthodontics and maxillofacial surgery, data from control groups are collected to act as a reference to objectively compare an individual or groups of patients. For example, lateral cephalograms [4] and more recently three-dimensional (3D) laser scans [5] from population groups can be age and/or sex matched, enabling comparisons to be made between an individual and their respective control template to guide treatment planning and measure outcome. Traditionally, assessment of lip function has been carried out using subjective grading scales [6] or descriptions of two-dimensional video recordings [7]. Advances in medical imaging have led to more sophisticated and objective measures of facial function being reported but their routine clinical application has been limited [8], [9]. Therefore, the aims of this study are to present a method/protocol for the analysis of lip shape during speech and to utilise statistical shape analyses to create an outcome measure for lip shape during speech for use in clinical interventions/rehabilitation.

## Materials and Methods

All participants provided their written consent to participate in this study. Ethical approval was obtained from South East Wales Research Ethics Committee (no. 09/41) prior to the commencement of the study. A sample participant consent form and information leaflet can be provided on request.

**Figure 1:**
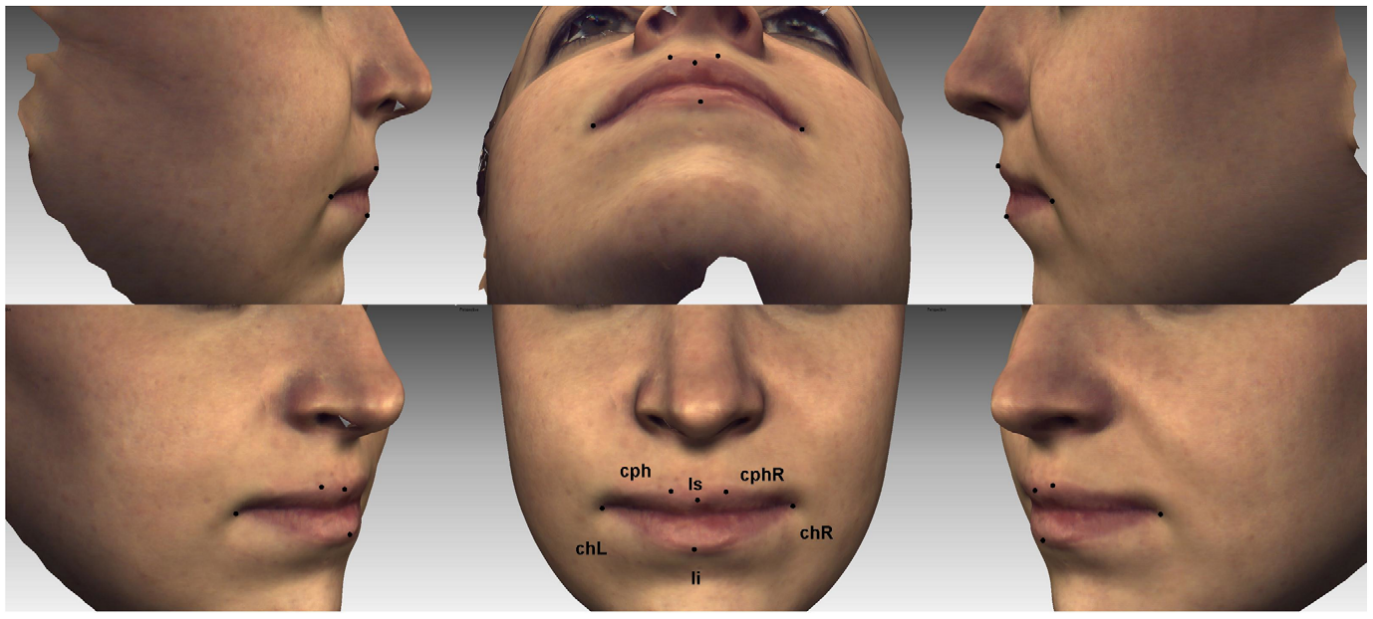
Lip landmarks used in study.

We confirm that the person in Figure 1 has seen this manuscript and figure and has provided written informed consent for their images to be used for publication.

### Participants

Inclusion criteria for the study were: the participants to be aged between 21–40 years, no relevant medical history, no history of facial surgery or paralysis, a full dentition with a Class 1 maxillary-mandibular skeletal relationship and British English as their first language. One hundred and fifteen white subjects (62 male, 53 female) with a mean age of 33.4 years were included.

Subjects were asked to say four verbal utterances (puppy, rope, baby, bob) in a normal, relaxed manner whilst scanned using the 3dMDFace™ Dynamic System (3Q Technologies, Atlanta, GA, USA) at 48 frames per second under standardised conditions. The system is a commercially available ultra-fast 3D surface scanner which captures images based on active stereophotogrammetry and uses a random infrared speckle projection to capture both pattern-projected and non-pattern projected white-light images simultaneously. The detailed specifications of the imaging system have been described in a previous publication [10].

### Image processing

The video sequences were analysed according to the visemes or mouth shapes for each word. The corresponding phonetic descriptions based on British English [11] are shown in Table 1. For the four words used in this study there are nine phonemes (including silence). As there is not always one-to-one mapping between phonemes and visemes - seven visemes (rest, **p**uppy, pupp**y**, **r**ope, **b**aby, bab**y**, b**o**b) were analysed in this part of the study. To account for temporal variations in the articulation of the visemes between subjects only the frame of maximal lip movement for each of the visemes was selected for analysis. This frame was selected by direct observation and represented the point at which the upper and lower lips were most apart in the vertical plane for the visemes **p**uppy and **b**aby, where the commissures were at their widest for the visemes pupp**y** and bab**y**, and where the lips were at their most protrusive for the visemes **r**ope and b**o**b.

**Table 1:** Phoneme to viseme mapping of the study words based on British English (visemes in bold).

Phoneme	Description	Viseme
/p/	Plosive consonant	**p**uppy
/b/		**b**aby
/i/	Long vowel	pupp**y**
		bab**y**
/r/	Approximant consonant	**r**ope
/<$>\raster="rg1"<$>/	Short vowel	b**o**b

Six landmarks were manually placed around the lips for the facial shell of maximum lip movement for each viseme (Figure 1). The landmarks are defined in anthropometric studies as: labiale superius (ls) - the midpoint of the upper vermilion line, labiale inferius (li) – the midpoint of the lower vermilion line, crista philtri (cph L/R) – the point on the left and right elevated margins of the philtrum above the vermilion line and cheilion (ch L/R) – the point located at the left and right labial commissure [12]. Following identification, the x, y, z coordinates of the 6 lip landmarks were recorded for each of the seven visemes. Closely matched maximal frames were all landmarked and the frame showing the greatest displacement vector for the particular viseme (as detailed above) was included in the analysis.

#### Landmarking error

Intra- and inter-examiner reproducibility of landmark placement has been previously assessed using mean distance error calculations [13]. The range of total landmark distance error for both intra- and inter-examiner assessments was 0.6–1.39 mm [14].

### Statistical analysis

Generalised Procrustes Analysis (GPA) was used to align the coordinates for all landmarks in the dataset. GPA is a rigid registration technique involving superimposition of landmark coordinates in optimal positions by means of their translation, rotation and scaling so as to minimise the sum of squared Euclidean distances [15]. Following registration, a centroid representing the mean position for the six landmarks for each of the seven visemes was derived. Two standard deviations (SD) around each centroid (representing 95% of the variability in *x*, *y*, and *z* from the mean) were calculated for all individuals and plotted as ellipsoids in RAPIDFORM™ software (INUS Technology Inc., Seoul, South Korea). This enabled the variation in lip shape for the visemes to be visualised individually. The mean displacement vectors from rest to maximal lip shape for each of the visemes were also tabulated to quantify the shape changes in the ellipsoid plots.

Canonical Variate Analysis (CVA) was then carried out using the peak x, y, z coordinates as predictor variables entered into the model for all seven visemes simultaneously. Viseme groupings were specified *a priori*. CVA projects multivariate data in a manner that maximises the separation between three or more given groups [16]. It is an extension of discriminant analysis and for *N* groups (in this example, seven viseme groups) will produce *N* – 1 axes (here, six canonical variates) of diminishing importance. Eigenvalues explain the amount of variation in lip shape for a particular canonical variate (CV). Significance testing of the CVs was conducted at a threshold of p<0.05 to provide a quantitative measure of which CVs statistically differentiated the visemes. CVA was carried out using SPSS 20.0.0 (SPSS Inc., Chicago, IL). Finally, the outcome measure for lip shape during speech was created by plotting the CV scores for each subject by viseme encompassed by 95% confidence intervals for those CVs that significantly differentiated the visemes.

### Data preparation

The use of CVA required the assumption of multivariate data normality and homogeneous variance-covariance matrices between groups to be satisfied [17]. Multivariate outliers were investigated using the Mahalanobis distance at a threshold of 42.3 [18]. There were five multivariate outliers identified outside the threshold that were removed prior to CVA. Homogeneity of variance-covariance matrices was tested using Box's M Test. This was violated (p<.001) and therefore separate-group covariance matrices were displayed for CVA.

## Results

**Figure 2:**
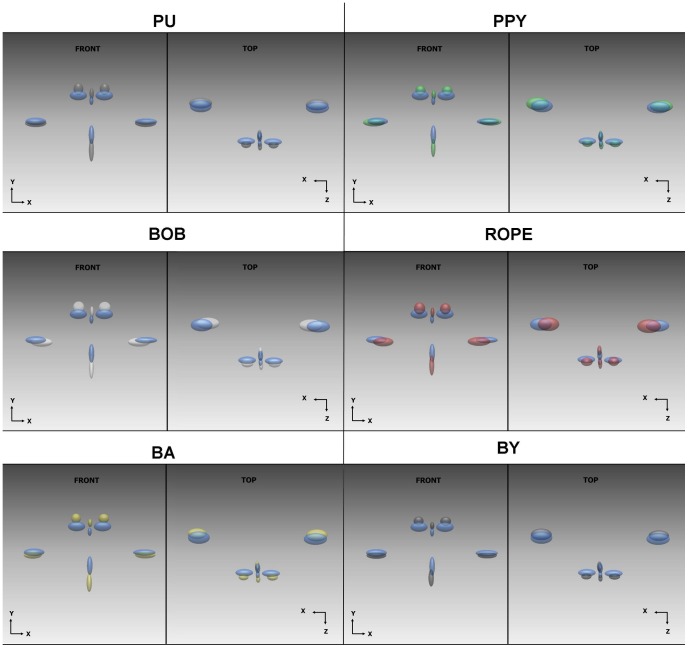
Ellipsoid plots showing variation in maximal lip movement from resting lip shape (blue).

Figure 2 shows ellipsoid plots of each viseme from the resting lip shape (blue) to peak amplitude. Tables 2, 3, 4, 5, 6, and 7 show the mean displacement vectors (in mm) for each of the visemes visualised in Figure 1. As maximum landmark reproducibility error has been previously been recorded at 1.39 mm (SD = 0.57) only mean displacements greater than 2.0 mm are considered as contributors to their respective visemes.

**Table 2:** Mean movement at peak amplitude for the viseme **p**uppy (bold highlights principal contributors >2.0 mm).

	x		y		z	
Landmark	Mean	SD	Mean	SD	Mean	SD
*ls*	−0.16	0.86	**−2.48**	2.32	**−2.29**	2.29
*li*	−0.28	1.20	**9.65**	3.59	−0.05	2.75
*cphL*	−0.22	2.46	**−3.05**	2.03	**−2.15**	2.08
*cphR*	0.01	2.43	**−3.46**	2.03	**−2.17**	2.18
*chL*	−0.34	2.62	−0.24	2.54	0.25	2.87
*chR*	0.23	2.34	−0.19	2.52	0.90	2.58

**Table 3:** Mean movement at peak amplitude for the viseme pupp**y** (bold highlights principal contributors >2.0 mm).

	x		y		z	
Landmark	Mean	SD	Mean	SD	Mean	SD
*ls*	−0.26	0.84	1.53	2.24	−1.36	2.19
*li*	−0.27	1.10	**7.63**	3.06	0.36	2.81
*cphL*	−0.47	2.34	1.17	2.20	−1.22	2.11
*cphR*	−0.08	2.13	1.26	2.20	−1.31	2.17
*chL*	−1.27	2.95	0.64	2.80	0.23	2.86
*chR*	1.77	2.77	0.98	2.56	0.85	3.06

**Table 4:** Mean movement at peak amplitude for the viseme rope (bold highlights principal contributors >2.0 mm).

	x		y		z	
Landmark	Mean	SD	Mean	SD	Mean	SD
*ls*	−0.19	1.06	−1.54	2.41	**−4.96**	3.35
*li*	−0.14	1.20	**7.55**	3.41	**−3.01**	3.59
*cphL*	−0.23	2.47	−1.02	2.29	**−4.49**	3.20
*cphR*	−0.11	2.56	−1.04	2.30	**−4.64**	3.22
*chL*	**3.68**	3.03	**2.29**	2.84	−1.31	4.68
*chR*	**−4.17**	2.69	**2.80**	3.16	−1.47	4.52

**Table 5:** Mean movement at peak amplitude for the viseme baby (bold highlights principal contributors >2.0 mm).

	x		y		z	
Landmark	Mean	SD	Mean	SD	Mean	SD
*ls*	−0.22	0.95	−1.90	2.26	−1.14	1.86
*li*	0.01	1.18	**9.06**	2.99	1.47	2.94
*cphL*	−0.58	1.92	−1.66	2.14	−0.92	1.86
*cphR*	0.11	1.82	−1.64	2.14	−1.03	1.88
*chL*	−1.45	2.37	**2.55**	2.62	**2.36**	2.86
*chR*	0.94	2.46	**2.68**	2.62	**2.54**	2.69

**Table 6:** Mean movement at peak amplitude for the viseme baby (bold highlights principal contributors >2.0 mm).

	x		y		z	
Landmark	Mean	SD	Mean	SD	Mean	SD
*ls*	−0.06	0.95	**−2.38**	2.16	−0.49	1.91
*li*	0.24	1.27	**5.50**	3.01	1.15	2.76
*cphL*	−0.36	1.96	**−2.07**	2.14	−0.37	1.86
*cphR*	0.11	1.78	**−2.13**	2.13	−0.42	1.90
*chL*	−1.06	2.56	1.35	2.65	**2.35**	2.65
*chR*	0.27	2.37	1.49	2.48	**2.34**	2.67

**Table 7:** Mean movement at peak amplitude for the viseme bob (red highlights principal contributors >2.0 mm).

	x		y		z	
Landmark	Mean	SD	Mean	SD	Mean	SD
*ls*	−0.26	1.04	−1.18	2.69	**−4.24**	2.90
*li*	0.01	1.10	**7.93**	3.81	**−2.28**	3.44
*cphL*	−0.16	2.01	−0.78	2.44	**−3.99**	2.85
*cphR*	−0.11	1.99	−0.80	2.46	**−3.95**	2.74
*chL*	**3.00**	2.88	**3.19**	2.73	**−3.31**	4.05
*chR*	**−3.01**	2.70	**3.40**	2.63	**−2.58**	3.82

The viseme **p**uppy can be described as principally a mean downward movement of the lower lip at *li* of up to 10 mm (Table 2). There is an associated mean upward movement of the midline, left and right upper lip at *ls* and *cph* of approximately 3 mm. This equates to an overall mouth opening of 13 mm. In addition to the vertical component, there is also a slight mean protrusive movement of the upper lip at *ls* and *cph* of up to 2.5 mm. There is negligible movement in the lateral plane. Except for a downward movement of the lower lip at *li* of 7.63 mm, there were no other mean landmark displacements that exceeded 2 mm for the viseme pupp**y** (Table 3).

The viseme **r**ope is principally composed of a downward movement of the lower lip at *li* with a mean of approximately 7.5 mm (Table 4). There is an associated mean downward movement of the left and right commissures of up to 3 mm. The commissures narrow the mouth aperture through medial movement of *chL* and *chR*. All landmarks show a mean protrusive element although this was primarily related to the upper lip. The magnitude of the standard deviation particularly in the Z plane suggests that there is a wide variation in protrusive movement for this viseme.

The principal mean movement for the viseme **b**aby is a downward movement of the lower lip in the order of 9 mm (Table 5). There is also a slight protrusive movement of the elevated margins of the upper lip. However this is only marginally above 2 mm in magnitude and the standard deviation suggests a high variation within the sample for these landmarks.

Mean landmark displacement for the viseme bab**y** involves a combination of downward movement of the lower lip and upward movement of the upper lip (Table 6). This is in favour of the lower lip in an almost 2∶1 ratio. There is also a protrusive element to the corners of the mouth, but in a similar finding to the preceding viseme, the magnitude is only marginally over 2 mm and as the standard deviation is relatively high, the variation in the sample is wide.

In a similar manner to **r**ope, the viseme b**o**b shows strong protrusive elements for all landmarks in the z plane (Table 7). In addition, there is contribution from *li* to mouth opening in the order of almost 8 mm. The corners of the mouth appear to move towards each other as well as moving downwards. Standard deviations for all principal contributors are relatively high implying a wider degree of variation in movement for the sample.

### Canonical variate analysis

**Figure 3:**
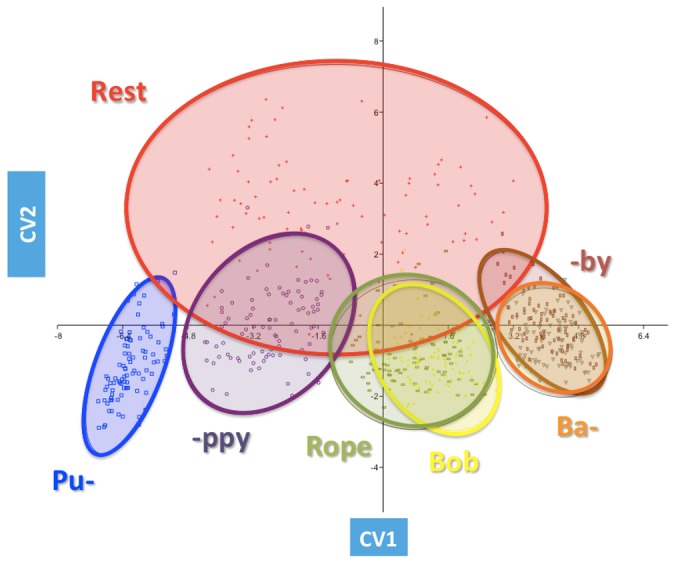
Scatterplot of CV Scores for CV1-2 labelled by viseme. Red cross = resting. Blue square = **p**uppy. Purple circle = pupp**y**. Yellow triangle = **r**ope. Green bar = **b**aby. Grey triangle = bab**y**. Brown bar = b**o**b.

Six CVs were revealed through the analysis with the first explaining 72.8% of the variance, whereas the second explained only 17.2% (Table 8). In total, the first two CVs accounted for 90% of the variance with CV3-6 explaining the remaining 10%. The significance of the model was tested as a whole, following which each variate was removed in turn to see whether the variates that remained were considered significant (Table 9). This showed that in combination, the first four CVs significantly discriminated the seven visemes. Despite this, scatterplots of the CV scores for each subject labeled by viseme showed that only CV1 and CV2 clearly differentiated the seven lip shapes (Figure 3). Encompassed by 95% confidence interval ellipses, the plot shows a wide variation in resting lip shape (red). A shift along the Y-axis (CV2) marks the change from resting lip shape to utterance articulation. Progression along the X-axis (CV1) differentiates the utterances. **P**uppy (blue) appeared to be the most distinct viseme whereas overlap of the 95% confidence interval ellipses suggested that **r**ope (brown) and b**o**b (yellow), and **b**aby (green) and bab**y** (grey) were extremely similar in peak lip shape.

**Table 8:** Summary of canonical variates.

CV	Eigenvalue	Variance (%)	Cumulative (%)
1	2.21	72.8	72.8
2	0.52	17.2	90.1
3	0.19	6.5	96.6
4	0.09	3.0	99.6
5	0.00	0.2	99.9
6	0.00	0.1	100.0

**Table 9:** Significance testing of canonical variates.

Test of Variates	Significance
1 through 6	0.00
2 through 6	0.00
3 through 6	0.00
4 through 6	0.00
5 through 6	0.98
6	0.95

The pooled within-groups correlations between the landmark coordinates and CVs are shown in Table 10. Coordinates are ordered by absolute size of their correlation within a CV. The largest absolute correlations between each coordinate and the first four CVs are highlighted. CV2, which explained 17.2% of the variance in the sample and was the variate that differentiated resting lip shape from articulation of the utterances and correlated with midline lip protrusion (*ls* Z and *li* Z) and vertical opening at the commissures (*chL* Y and *chR* Y). CV1, which explained 72.8% of the variance, differentiated between the difference visemes and correlated with changes in vertical mouth opening (*ls* Y, *li* Y, *cphL* Y and *cphR* Y) and mouth width (*chL* X and *chR* X).

**Table 10:** Correlations between landmark coordinates and CVs (significant CVs in bold).

Coordinate	CV					
	1	2	3	4	5	6
ls Y	**0.76**	−0.23	−0.28	−0.01	−0.03	−0.21
li Y	**−0.70**	0.01	0.51	0.30	0.10	0.16
cphL Y	**0.67**	−0.38	−0.17	−0.26	−0.02	−0.09
cphR Y	**0.66**	−0.35	−0.22	−0.12	−0.27	0.11
chR X	**0.51**	−0.50	0.08	−0.24	−0.35	−0.40
chL X	**−0.50**	0.46	0.38	0.30	−0.12	−0.25
chL Y	−0.25	**0.52**	−0.35	−0.02	0.21	0.05
ls Z	0.34	**−0.50**	0.16	0.04	−0.24	0.01
chR Y	−0.29	**0.47**	−0.15	−0.28	−0.11	−0.23
cphR Z	0.41	**−0.45**	0.41	0.11	0.21	0.23
li Z	−0.27	**0.35**	0.32	−0.30	0.04	0.12
cphR X	0.10	**0.28**	−0.04	−0.10	0.04	0.08
chL Z	−0.11	0.14	**−0.78**	0.22	0.27	0.08
cphL Z	0.26	−0.30	**0.53**	−0.24	−0.13	0.17
li X	−0.04	−0.02	−0.35	**−0.48**	−0.01	0.45
ls X	0.06	−0.04	0.27	−0.06	0.66	0.41
chR Z	−0.16	.016	−0.32	0.22	−0.21	−0.50
cphL X	−0.03	−0.26	−0.24	0.26	0.24	0.29

## Discussion

In this study, a sample of 115 *average* subjects was used to model ordinary lip movement for different visemes. When reviewing the literature for databases that have used 3D data to construct profiles of average facial movement, a benchmark of approximately 100 subjects has been quoted [19], [20]. In this respect, the number of participants recruited can be considered acceptable.

The 115 subjects were asked to say four utterance or verbal gestures. Many previous studies have utilised non-verbal gestures such as facial expressions as a measure of lip/facial movement [21], [22]. Clinically, the facial gesture that is used should be reproducible over time so that it is performed as near to the same way each occasion with as little variation as possible. In this respect, the effect of a clinical intervention on facial movement can be truly quantified. Previous research suggests that verbal facial gestures are more reproducible over time than non-verbal [23] and therefore verbal gestures were adopted for this study. Furthermore, the words chosen are bilabial speech postures [24] stimulating the lip articulators and have a clinical connotation being used in cleft speech assessments [25].

Only the maximum frame of lip movement was analysed in this study partly due to temporal variations in the articulation of visemes between the subjects. In addition, the time required to manually landmark all the facial shells in a sequence would result in several thousand images to process, which was considered unfeasible. Therefore peak lip shape was considered as a comparable point in time across the sample. Clearly the choice of maximal frame could influence the outcome of the results and the reliability of choosing this frame was not investigated. However, given the relatively high frame capture rate of 48 frames per second, a one-frame discrepancy is unlikely to skew the results significantly [26]. Another aspect of only choosing the maximal frame for analysis is that information on speech and trajectory of the visemes is unavailable. As automated methods of image registration and landmark identification/tracking are developed, the capacity for larger volumes of data to be analysed will increase [27].

Once the x, y, z data from the facial shells had been extracted, GPA ensured that all coordinates were aligned in the same 3D space, which compensated for head movements during articulation. Other studies have used head frames to introduce immobile reference points to compensate for head movements [28], but using GPA eliminates this requirement. The other advantage of the GMM approach is that the coordinates of the landmarks are statistically analysed rather than inter-landmark distances. This allows the results of the statistical analyses to be visualised as deformations of landmark configurations thereby increasing the sensitivity as more shape information is analysed [29].

The CVA model showed that the visemes **r**ope and b**o**b, and **b**aby and bab**y** were essentially the same (showing concentric 95% confidence intervals), and therefore from a clinical perspective only the most reproducible visemes could be retained. The clear separation of the visemes along CV1 represents potential for use as a clinical outcome measure. Data from a single patient or patient groups can be analysed in a similar manner to the GMM approach described and projected onto the average CVA model. Abnormal lip movement could be identified and indeed quantified by the distance of the patient data from the 95% confidence interval of the average model thereby acting as a diagnostic tool during clinical examinations and as a functional outcome measure following an intervention/rehabilitation. Despite basing the clinical model on verbal utterances implying that the data is specific to the geographical area and language it could also act as a template to compare lip shape/movement from different populations.

## Conclusion

An average model has been created for lip shape during movement through two canonical variates; one of which distinguishes resting lip shape from the four utterances and the other discriminates between the four utterances. The method utilises pre-existing statistical shape analysis and can be reproduced in the clinical setting to provide a diagnostic and functional outcome tool.
